# Decoding the distinct immune landscape and possible regulatory mechanisms of autoimmune hepatitis through integrated single-cell and bulk RNA sequencing

**DOI:** 10.1371/journal.pone.0335605

**Published:** 2025-12-04

**Authors:** Gang Chi, Yijia Che, Jinhong Pei, Xueqing Li, Junmei Wang

**Affiliations:** Department of Biochemistry, Changzhi Medical College, Changzhi, Shanxi, China; University Hospital of Bologna Sant'Orsola-Malpighi Polyclinic Department of Digestive System: Azienda Ospedaliero-Universitaria di Bologna Policlinico Sant'Orsola-Malpighi Dipartimento dell'apparato digerente, ITALY

## Abstract

**Objectives:**

Autoimmune hepatitis (AIH) is a complex immune-mediated liver disorder characterized by dysregulated immune responses. This study aimed to decode the distinct immune landscape and regulatory mechanisms of AIH using integrated single-cell and bulk RNA sequencing.

**Methods:**

The data of single-cell RNA-seq (scRNA-seq) and bulk RNA-seq were downloaded from GEO database. The cell clustering, differential gene expression, trajectory analysis, functional enrichment, and cell-cell communication were performed were analyzed using R software.

**Results:**

Five major immune cell types were identified, with CD8 + T cells and NK cells significantly expanded in AIH. Functional enrichment showed upregulation of immune activation, inflammation, and metabolic pathways in these cells. The cell-cell communication analysis revealed robust interactions between CD8 + T and NK cells, primarily driven by the CCL5-CCR signaling axis. Integrative analysis of scRNA-seq and bulk RNA-seq identified four common DEIRGs (ITK, IL7R, CXCR4 and SORT1) and one transcription factor PRDM1.

**Conclusion:**

This study identified dysregulated immune cell clusters, signaling pathways, and potential therapeutic targets in AIH.

## Introduction

Autoimmune hepatitis (AIH) is a chronic, immune-mediated liver disease characterized by hepatocyte inflammation, progressive fibrosis, and liver failure if untreated. Despite reducing liver inflammation and preventing disease progression, immunosuppressive therapy carries potential side effects and a risk of relapse upon discontinuation, indicating that current treatments fail to restore intrahepatic immune regulation [1,2]. Increasing the functional activity of hepatic immune cells may help reinstate immune regulatory balance within the liver. However, the hepatic immune microenvironment in AIH is a highly intricate network, characterized by a complex immune landscape with extensive immune cellular heterogeneity. It remains unclear how the heterogeneity of these immune cell types, their functional states, and the underlying molecular mechanisms contribute to immune dysregulation.

Despite AIH being typically associated with autoreactive T cell activation, B cell overactivity, and enhanced autoantibody production [3,4], the precise regulatory mechanisms underlying immune dysfunction in this disease remain poorly understood. Recent studies have revealed that CTLA-4 deficiency in mice resulted in lethal autoimmune hepatitis characterized by lymphocyte infiltration in liver [4]. TGFB1 deficiency exacerbated immune reactions but impaired metabolic activity in AIH [5]. However, the specific immune-related gene networks and their regulatory mechanisms in AIH remain largely unexplored. A deeper understanding of these mechanisms is essential for elucidating the drivers of immune dysregulation and identifying novel therapeutic target.

Recent advances in high-throughput sequencing technologies, particularly single-cell RNA sequencing (scRNA-seq) and bulk RNA sequencing, offer powerful tools for dissecting the cellular and molecular complexity of immune-mediated diseases. scRNA-seq provides high-resolution insights into the cellular diversity and dynamic cellular communication changes by capturing transcriptomic profiles at the single-cell level, enabling the precise identification of distinct immune cell clusters and their functional states [6,7]. Bulk RNA sequencing offers a broader perspective on global gene expression changes across patient cohorts, facilitating the identification of disease-associated molecular signatures [8].

In this study, we combined scRNA-seq and bulk RNA-seq analyses to characterize the distinct immune landscape of AIH. The objective was to identify dysregulated immune cell clusters, signaling pathways, and potential therapeutic targets associated with AIH progression. The findings of this study provide novel insights into the immunopathogenesis of AIH and may contribute to the development of more targeted therapeutic strategies for this complex autoimmune liver disease.

## Materials and methods

### Single-cell transcriptomic data analysis

Single-cell RNA-seq (scRNA-seq) data (GSE216064) of PBMCs for 4 autoimmune hepatitis patients (AIH) and 4 healthy controls (HC) were downloaded from Gene Expression Omnibus (GEO). All the samples were processed with 10 × Genomics Gene Expression 3’ Chromium V 2.0 per the manufacturer’s instructions [9]. Low-quality cells with less than 500 or over 4000 expression genes, or high number of mitochondrial genes (>20%) were filtered out from further analysis [10]. Filtered cells were standardized by NormalizeData function and 2000 most variable genes were identified using FindVariableFeatures function in Seurat package. The principal component analysis (PCA) was then performed on the variable genes. Uniform manifold approximation and projection (UMAP) dimension reduction was performed based on the top 15 significant principal components (PCs). The harmony (V1.2.1) was used to remove batch effects between the samples. Cell clusters were identified using FindNeighbors and FindClusters functions at a resolution of 0.2. The FindAllMarkers function with min.pct of 0.25 and logfc.threshold of 0.25 was used to find marker genes in various clusters or identify differentially expressed genes (DEGs) for each cluster. The R package SingleR was used to evaluate the cell type annotation results [11]. The cell type distribution in AIH and HC groups was calculated to find the differential cell types. All analyses were performed using R software (version 4.4.1) and Seurat package (version 5.1.0).

### Bulk transcriptomic data processing

Bulk transcriptomic data of GSE159676 and GSE206364 were available from the GEO database. GSE159676 comprised 3 AIH patients and 6 HC patients for liver tissues and GSE206364 included 5 pediatric autoimmune hepatitis and 9 pediatric healthy controls for liver tissues [12,13]. Raw data was downloaded using the GEOquery package [14] and DEGs were identified using the limma package [15]. The Bulk DEG were determined by intersecting the most significant DEGs between GSE159676 and GSE206364 datasets (adjusted P value <0.05, and absolute logFoldChange > 0.3).

### Heterogeneity assessment

The cluster purity was assessed using the ROGUE package [16] with default parameters, following recommended pipelines.

### Gene set variation analysis and functional enrichment analysis

Human gene sets from 50 hallmark pathways were retrieved using msigdbr package. GSVA package was used assess the functional differences in the corresponding cell populations [17]. The GO and KEGG pathways were analyzed using the ClusterProfiler package. The ggplot2 package was conducted for visualization.

### Differentially expressed immunologically relevant gene (DEIRG) score

A curated list of immunologically relevant genes was obtained from ImmPort [18]. The DEIRGs was obtained by intersecting this list with the DEGs and the DEIRG score was then calculated using the AUCell package [19].

### Trajectory analysis

Single-cell pseudotime trajectories were calculated and displayed based on monocle3 package [20]. The data were preprocessed using the PCA method, and dimensionality reduction was performed with the uniform manifold approximation and projection (UMAP) method.

### Cell-cell communication analysis

The intercellular communication analysis was conducted using the CellChat package [21], which provided a curated repository of ligand-receptor and transcription factor interactions as well as a statistical framework for inferring lineage-specific interactions.

### Common DEIRGs and transcription factors regulatory mechanisms

The common DEIRGs was obtained from differentially expressed genes of immune cell clusters and immune-related genes (IRGs). The human transcription factors were downloaded from hTFtarget [22] and AnimalTFDB (version 4.0) [23]. The differentially expressed transcription factors (DETFs) was obtained from the differential cell types clusters and Bulk DEGs.

### Ethics statement

All data used in the study is publicly available, hence no ethical approval was needed.

## Results

### Single-cell transcriptome profiles and functional states in AIH

**Fig 1:**
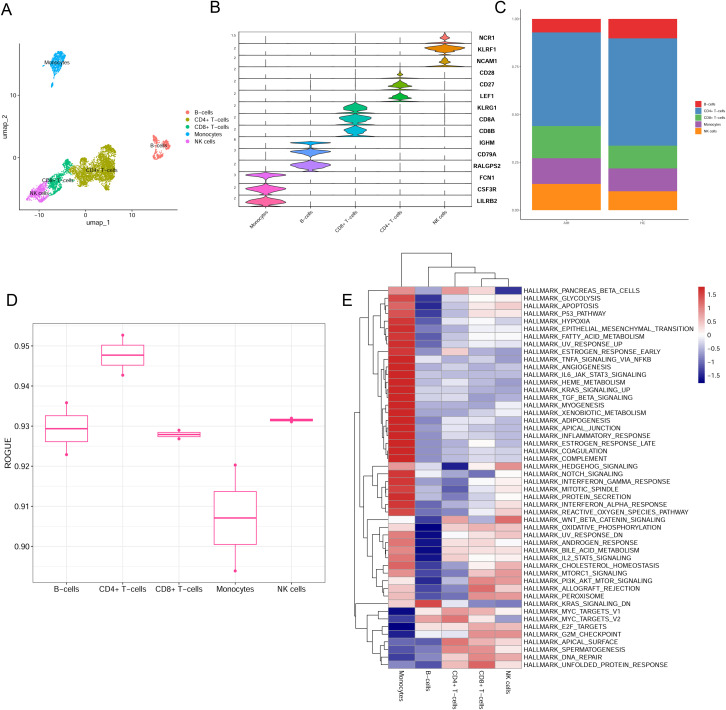
Immune landscape and molecular features of of immune cell clusters in AIH. **(A)** UMAP projection displayed major immune cell clusters from PBMC of AIH and unique colors highlight each cell type. **(B)** Violin plots displaying expression distribution of key marker genes across different immune cell types. **(C)** Bar plot showing the proportional distribution of immune cell clusters between the AIH and control groups. **(D)** ROGUE-based cell purity assessment for each cluster. **(E)** GSVA analysis in the corresponding immune cell clusters.

The single-cell profiling of immune cells in AIH liver tissues and healthy controls was obtained from GSE216064, and five major immune cell types were identified, including B cells, CD4 + T cells, CD8 + T cells, monocytes, and NK cells (Fig 1A and S1 Fig in S1 File). These cell types were further validated using canonical marker genes, demonstrating high specificity and purity (Fig 1B). The distribution of immune cells exhibited significant alterations, with CD8 + T cells and NK cells showing a marked increase in AIH samples, suggesting their potential involvement in disease pathogenesis (Fig 1C). Moreover, ROGUE scores were used to evaluate the purity and heterogeneity of these immune cell types, which revealed that B cells, CD4 + T cells, CD8 + T cells and NK cells exhibited higher purity, whereas monocytes showed greater heterogeneity (Fig 1D), indicating diverse functional states within these immune cell populations. To further explore potential regulatory mechanisms underlying the altered immune environment in AIH, hallmark gene sets were used to investigate pathway activity. Strikingly, monocytes, CD8 + T cells and NK cells showed marked upregulation of pathways related to immune activation, inflammation, metabolic reprogramming and cellular signaling (Fig 1E), indicating that the immune dysregulation in AIH may be driven by specific functional states of these immune cells.

### Single-cell differential expression and functional enrichment analysis in AIH

**Fig 2:**
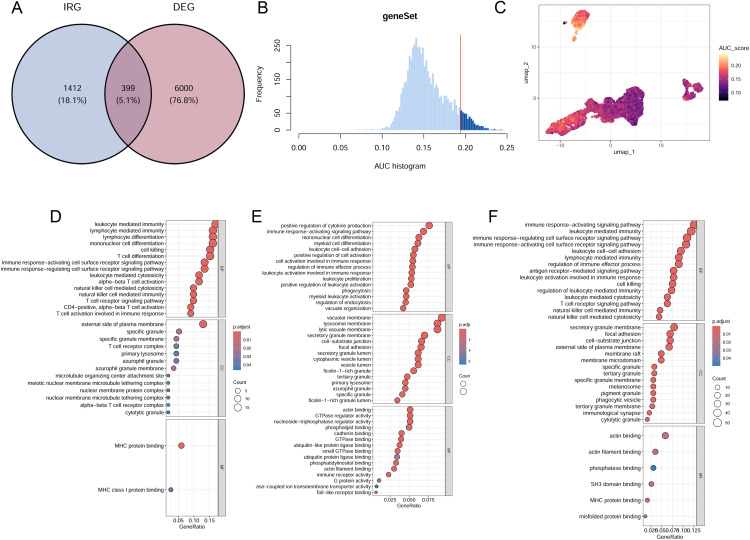
Single-cell differential expression and functional enrichment analysis in AIH. **(A)** Venn diagram of screened DEIRGs from immune-related genes (IRGs) and differentially expressed genes of immune cell clusters. **(B)** Score of 399 screened DEIRGs. The threshold was 0.194. **(C)** UMAP plots of DEIRG score in immune cell clusters. **(D-F)** GO enrichment analysis of upregulated DEGs in CD8 + T cells **(D)**, monocytes **(E)** and NK cells **(F)**.

To further understand the molecular mechanisms underlying the observed immune cell dysregulation, we performed differential gene expression analysis across the identified immune cell clusters. We identified 399 differentially expressed immune-related genes (DEIRGs) from a total of 6,399 differentially expressed genes (Fig 2A). To further explore the functional relevance of these DEIRGs, we evaluated their activity levels across different immune cells (Fig 2B). The analysis revealed that CD8 + T cells, monocytes and NK cells exhibited higher activity scores and expressed more genes, suggesting their pivotal role in the immune dysregulation during AIH development (Fig 2C). Given the significant increase of these immune cells in AIH (Fig 1C), we further conducted functional enrichment analyses of DEGs within the CD8 + T cell, monocyte, and NK cells. These analysis revealed the top 15 upregulated genes in CD8 + T, monocytes and NK cells were associated with antigen processing and immune response (Fig 2D-2F). Additionally, the downregulated genes in CD8 + T, monocytes and NK cells were mostly related with immune response and energy metabolism (S2 Fig in S1 File).

### Characterization of single-cell trajectory analysis and cell–cell communication in AIH

**Fig 3:**
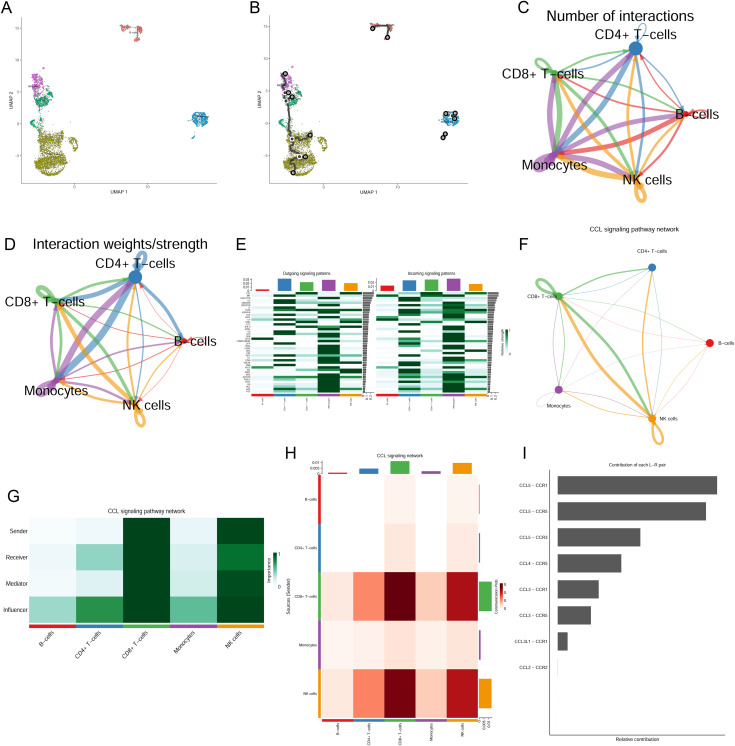
Single-cell trajectory and communication networks analysis in AIH. **(A)** UMAP visualization of immune cell heterogeneity. **(B)** Two-dimensional cell trajectory analysis. **(C)** Number of interactions. The peripheral circles of different colors represented the number of cells, with larger circles indicating a higher cell count. The thicker lines indicated a greater number of ligand-receptor pairs. **(D)** Interaction weights/strength illustrating the cumulative strength of intercellular communications. **(E)** Heatmap showing outgoing and incoming signaling patterns for each immune cell cluster. **(F)** Inferred CCL signalling pathway network. **(G)** Relative importance of immune cell clusters in the CCL signaling network according to the computed four network centrality measures. **(H)** Heatmap representation of the sender–receiver communication probability in the CCL signaling network, with signal intensity indicated by color shade. **(I)** Relative contribution of each ligand-receptor pairs to the overall CCL signaling network.

To explore the immune landscape in AIH, we performed single-cell trajectory analysis to reconstruct the differentiation pathways of immune cells. We reconstructed the developmental relationships among immune cells, revealing key differentiation stages and transition states (Fig 3A, 3B and S3A Fig in S1 File). We further performed comprehensive cell-cell interaction analysis to characterize the communication networks. Both the number and strength of interactions among immune cells were high, revealing extensive cross-talk, particularly involving elevated CD8 + T cells and NK cells in AIH, which exhibited an increased interaction (Fig 3C, 3D and S3B-S3D Fig in S1 File). Among the identified significant communication pathways, the C-C Motif Chemokine Ligand (CCL) signaling pathway emerged as the dominant mechanism in CD8 + T cells and NK cells (Fig 3E). Notably, the CD8 + T-cell and NK cell cluster showed the largest number of ligand-receptor pairs directed towards the monocyte cluster (S3E Fig in S1 File). Furthermore, particularly strong interactions between CD8 + T cells and NK cells were identified within the CCL signaling pathway network (Fig 3F-3H). The most significant ligand-receptor pair identified was CCL5-CCR1, followed by CCL5-CCR5 and CCL5-CCR3 (Fig 3I and S3F Fig in S1 File), suggesting a predominant role for CCL5-mediated signaling in orchestrating the immune cells cross-talk, especially involving the CD8 + T-cell and NK cell clusters.

### Transcriptomic analysis of regulatory mechanisms in AIH

**Fig 4:**
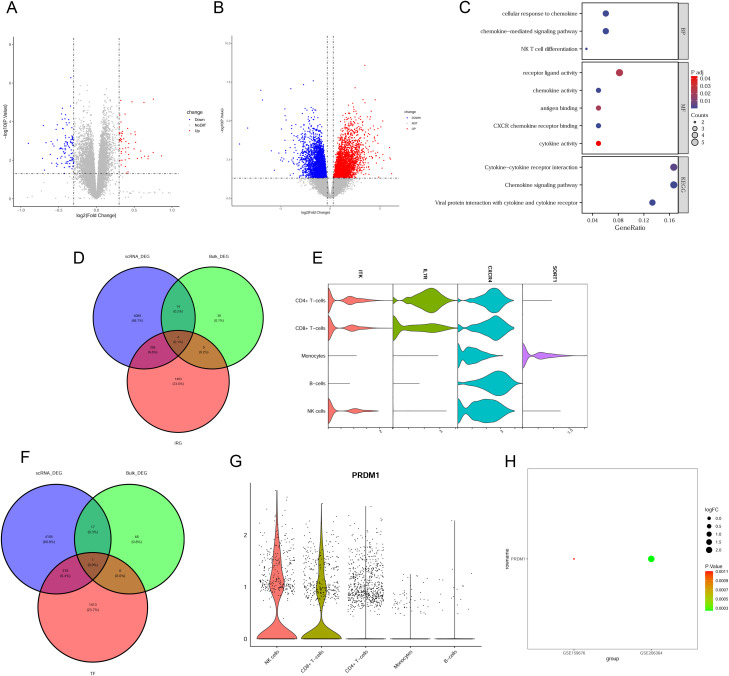
The regulatory mechanisms in Bulk DEGs of AIH liver tissues, DEIRGs and transcription factors. **(A)** Volcano plot of DEGs in the GSE159676 dataset. **(B)** Volcano plot of DEGs in the GSE206364 dataset. **(C)** GO and KEGG enrichment analysis of bulk DEGs. **(D)** Venn plot showed the common DEIRGs between scRNA DEGs and bulk DEGs. **(E)** Stacked violin plot of common DEIRGs across immune cells from PBMC of AIH. **(F)** Venn plot showed the common DETFs between scRNA DEGs and bulk DEGs. **(G)** Stacked violin plot of one common DETF (PRDM1) in immune cells from PBMC of AIH. **(H)** Bubble plot of one common DETF (PRDM1) expression in GSE159676 and GSE206364 dataset.

To further validate the single-cell findings, we performed integrative analysis using bulk transcriptomic data from AIH liver tissue. We identified a total of 166 and 6126 DEGs in GSE159676 and GSE206364 in human liver tissue, respectively (Fig 4A, 4B). Functional enrichment analysis of the intersecting DEGs highlighted immune-related pathways, including cytokine activity, chemokine signaling, and NK cell differentiation (Fig 4C), which supported the expansion of NK cells and CD8 + T cells in the peripheral blood of AIH patients. Given the prominence of immune-related pathways in both the single-cell and bulk transcriptomic data, we further explored the common expression patterns of DEIRGs between different immune cell clusters from PBMC of AIH and liver tissue samples. Four common DEIRGs (ITK, IL7R, CXCR4 and SORT1) were shared between the scRNA-seq immune cell clusters and the bulk liver tissue samples (Fig 4D). These shared DEIRGs exhibited notable dysregulation across different immune cell clusters from PBMC of AIH (Fig 4E and S4A Fig **in** **S1 File**). Moreover, the expression of IFN-γ, cytotoxic mediators and CCL5 were significantly upregulated in AIH patients compared with healthy controls (S4B Fig **in** **S1 File**). The role of transcription factors in regulating the immune response was investigated. Only one common differentially expressed transcription factor (DETF) was found to be dysregulated in both the scRNA-seq immune cell clusters and the bulk liver tissue samples (Fig 4F). This transcription factor, PRDM1, showed significantly upregulation in NK and CD8 + T cell clusters from PBMC of AIH compared to other immune cell clusters (Fig 4G and S4C Fig **in** **S1 File**). However, PRDM1 was significantly upregulated in GSE206364 while significantly downregulated in GSE159676 (Fig 4H).These findings suggested a potential regulatory mechanism involving PRDM1 in the immune response of AIH.

## Discussion

In the present study, we analyzed scRNA-seq and bulk RNA-seq data for AIH and presented the transcriptional and regulatory landscape of AIH. The expansion of CD8 + T cells and NK cells in AIH is intricately linked to immune activation. The CCL5-CCR signaling axis played a crucial role in the cross-talk between CD8 + T cells and NK cells. We identified four shared DEIRGs (ITK, IL7R, CXCR4 and SORT1) and identified PRDM1 as a potential transcriptional regulator of immune dysregulation in AIH.

Our single-cell RNA analysis revealed a distinct immune landscape in AIH characterized by significant expansion of CD8 + T cells and NK cells, as well as the functional heterogeneity of monocytes. It was previously shown that CD8 + T cells as the predominant population in interface lymphocytic infiltration in active AIH, while CD4 + T cells primarily localized to the central portal tract area [24]. The expanded CD8 + T cell population in our study exhibited upregulated expression of cytotoxic mediators (perforin and granzyme B), consistent with prior findings that elevated levels of these cytotoxic effectors in AIH patients compared to healthy individuals [4]. Although CD4 + T cells also played a key role in AIH pathogenesis, their reduced presence in our study may be due to immunosuppressive treatment (prednisolone and azathioprine) in AIH patients from GSE216064 data. Thus, our findings showed the immune landscape during active immunosuppression rather than treatment-naive disease states. It has been demonstrated that CD4 + T cells do not cause hepatitis in the absence of CD8 + T cells and are less prone to activation by liver antigens under non-inflammatory conditions [25]. The immunosuppressive treatment likely suppressed CD4 + T cell numbers while allowing CD8 + T cells to persist in an activated state. Studies have revealed that persistent immune activation and autoimmunity at the cellular level, even during effective immunosuppressive treatment and disease remission [26]. Similarly, the expansion of NK cells in AIH liver tissues observed in our study was consistent with findings that NK cells could directly attack liver parenchyma or contribute to damage through cytokine secretion and cell-to-cell contact [27]. The functional enrichment analysis in our study also revealed upregulation of genes involved in immune activation and inflammation in NK cells. This previous study demonstrated that NK cells secrete various cytokines and chemokines such as IFN-γ, TNF-α and CCL5, contributing to the progression of autoimmune diseases by stimulating autoreactive T and B cells [28]. It was also found that NK cells in AIH patients expressed significantly higher levels of IFN-γ, CCL5, perforin, and granzyme B compared to the healthy control in our study. This increased cytokine production likely reflected the functional activation of NK cells in the AIH microenvironment. These findings may explain the continued expansion and activation of CD8 + T cells and NK cells observed in our analysis. The differential susceptibility of immune cell subsets to immunosuppressive agents may create a skewed immune profile that, while clinically relevant for treated patients, may not fully represent the untreated disease pathophysiology.

The cell-cell communication analysis in this study demonstrated robust interactions between CD8 + T cells and NK cells, mediated predominantly through the CCL5-CCR1 axis, followed by CCL5-CCR5 and CCL5-CCR3. CCL5 was well-established as a potent chemoattractant that induced migration and recruitment of T cells, NK cells, and other immune cells to sites of inflammation through interaction with its receptors [29]. It has been established that CCL5 bound CCR1 and CCR5 with higher affinity than CCR3 [30], explaining the predominance of CCL5-CCR1/CCR5 interaction observed in our analysis. Thus, the expansion of CD8 + T cells and NK cells in AIH samples, combined with their intensified CCL5-mediated crosstalk in this study, suggesting this signaling axis may drive disease development. CD8 + T cells are recognized as potent producers of CCL5, secreting higher levels than CD4 + T cells [31]. CCL5 production was particularly associated with effector CD8 + T cells and could be triggered by activation with autoantigens in primary sjogren’s syndrome [31,32], suggesting this mechanism may similarly be occurring in AIH. Moreover, CCL5-CCR5 signaling provided resistance to apoptosis in T cells while enhancing T cell proliferation and cytokine production [33], potentially contributing to the persistent immune activation observed in AIH, even following immunosuppressive therapy in this study. Previous study also found that CCR5 was essential for NK cell accumulation in the liver and CCR5-deficient mice exhibit drastically reduced NK cell frequency [34,35]. Similarly, CCR1 blockade reduced renal infiltration of T cells and mononuclear phagocytes in lupus nephritis [36]. Thus, CCL5-mediated signaling may represent a conserved mechanism across autoimmune diseases, highlighting its therapeutic potential.

Our integrative analysis of single-cell and bulk RNA-seq data identified PRDM1 (encoding Blimp-1) as the only common differentially expressed transcription factor across immune cell clusters and liver tissue samples, suggesting its central role in AIH pathogenesis. Intriguingly, PRDM1 exhibited distinct regulation patterns depending on cellular context – markedly upregulated in peripheral blood NK and CD8 + T cell clusters but displaying opposing trends in liver tissue samples (upregulated in GSE206364 vs downregulated in GSE159676), suggesting cell-type- and tissue-specific regulatory mechanisms. This apparent contradiction likely reflected the dual functionality of PRDM1 as both a transcriptional repressor and activator [37]. It has been showed that PRDM1 acted as a transcriptional repressor in B cell terminal differentiation but functioned as an activator in NK and CD8 + T cells by regulating terminal differentiation and functional maturation of these cell types [38,39]. The repressive function of PRDM1 could attenuate liver inflammation [40], whereas its activation in circulating immune cells may sustain systemic immune activation. Moreover, PRDM1 regulated granzyme B production and chemotaxis/adhesion molecules (CCL3, CCL5, and CCR5) in NK and CD8 + T cells [41–43]. Consistent with our findings of elevated granzyme B and CCL5-CCR5 signaling, these results supported their role in driving immune activation and inflammation in AIH. Thus, PRDM1 served as a transcriptional rheostat, with low expression sustained T cell function but high expression driving T cell exhaustion [44]. The four shared DEIRGs (ITK, IL7R, CXCR4, SORT1) further underscored the interaction between effector activation and tissue homing. CXCR4 and IL7R were critical for lymphocyte trafficking and survival, while ITK modulated T-cell receptor signaling [45–47]. Their dysregulation in both PBMCs and liver tissue suggested the circulating immune cells may infiltrate the liver, exacerbating inflammation. Notably, CCL5-CCR axis-driven crosstalk between CD8 + T and NK cells may amplify this process.

However, AIH exhibited clinical heterogeneity that significantly impacted immune profiles and treatment responses. Patients presented with diverse clinical phenotypes ranging from acute hepatitis to established cirrhosis or asymptomatic chronic liver disease, each potentially harboring distinct immune signatures [48]. Acute hepatitis occurred in approximately 25% of AIH cases; these presentations represented spontaneous exacerbations of pre-existing chronic disease, newly developed disease, or acute disease triggered by various factors [49,50]. Our findings of robust CD8 + T cell and NK cell activation align with the immunological storm characteristic of acute presentations. Moreover, patients with cirrhosis experienced markedly worse outcomes, with 10-year survival rates of only 61.9%, compared to 94.0% in patients without cirrhosis [51]. The persistent activation of CD8 + T and NK cells observed in our study represented a pathological shift from protective immunity to tissue-destructive inflammation. Previous studies showed that CCL5 significantly influenced the migration and proliferation of stellate cells [18,52]. Consequently, the enhancement of the CCL5-CCR pathway in our analysis may have driven ongoing hepatic stellate cell activation and collagen deposition, perpetuating the fibrotic process even in the presence of immunosuppressive therapy. Notably, approximately 25% to 33% of patients with AIH were asymptomatic [48]. Despite the absence of symptoms, these patients exhibited histological features remarkably similar to those of symptomatic patients with AIH [53]. Alarmingly, about 26% to 70% of asymptomatic patients developed symptoms during follow-up [51,53]. Thus, our transcriptomic evidence of ongoing immune activation in patients with AIH provided a molecular basis for the lower 10-year survival rate in untreated mild cases of AIH [54].

The clinical heterogeneity of AIH was influenced by patient age, serving as a key determinant of disease characteristics and outcomes. Elderly AIH patients typically exhibited more frequent positivity for antinuclear autoantibodies, stronger associations with HLA-DR4, and a higher prevalence of concurrent autoimmune thyroid disorders compared to younger patients [55]. Furthermore, significant age-related HLA distribution patterns existed, with HLA-DRB1*04 occurring more frequently in elderly patients than in younger adults, while HLA-DRB1*03 predominated in younger patients [56]. These genetic differences may partially explain the clinical heterogeneity observed across age groups. Our molecular findings also provided potential mechanistic insights into this age-related diversity. The predominant activation of CD8 + T cells and expansion of NK cells in our study may vary significantly across different patient age groups due to age-associated immune dysfunction (immunosenescence). Immunosenescence paradoxically predisposed elderly individuals to exaggerated inflammation, autoimmune response, and immunodeficiency, resulting in significantly higher serum IgG levels and antinuclear antibody titers, despite lower hepatitis activity scores in elderly AIH patients [57,58]. Moreover, the enhanced CCL5-CCR signaling pathway identified in our analysis may contribute differentially to disease pathogenesis across age groups, particularly given the significant epidemiological shift toward older age at presentation. Elderly-onset AIH patients (≥70 years) comprised 37% of total AIH cases, followed by patients in their 60s (25%) [57]. This suggested that immune dysregulation associated with aging may be more strongly linked to AIH development than genetic predispositions in elderly populations [57].

The clinical heterogeneity of AIH also involved distinct autoimmune phenotypes characterized by unique serological and genetic profiles, which may substantially influence the age-related differences observed. Type 1 AIH (AIH-1) was characterized by antinuclear antibody (ANA) and/or smooth muscle antibody (SMA), whereas type 2 (AIH-2) was defined by anti-liver kidney microsomal type 1 (anti-LKM1) and/or anti-liver cytosol type 1 (anti-LC1) antibodies [59,60]. AIH-1 predominantly affected adults, while AIH-2 was more prevalent in pediatric populations. Notably, patients with AIH-2 presented more acutely, with significantly higher bilirubin and transaminase levels [61]. Moreover, the genetic landscape highlighted this heterogeneity, with distinct HLA associations influencing disease susceptibility and potentially immune response patterns among populations. Geographic and ethnic variations in genetic susceptibility patterns were revealed, with the B8-DR3-DQ2 phenotype being significantly more common in Italian type 1 AIH patients. In contrast, HLA DR4 – an independent risk factor in North America and Northern Europe – showed no association with type 1 or type 2 AIH in Italy [62]. HLA DR3 and B8-DR3-DQ2, the primary genetic risk factors for AIH in North America, were less common in Italy. The genetic susceptibility to AIH in Italy differed from that in North America, indicating the involvement of additional genetic or etiological factors [62]. This difference may be functionally significant, as distinct HLA alleles present unique peptide repertoires to CD4 + T cells, potentially explaining variations in disease severity, autoantibody profiles, and treatment responses [61]. In AIH-2, HLA-DRB1*0701-positive patients exhibited CD4 + /CD8 + T cell cross-reactivity to five of seven CYP2D6 epitopes, with elevated frequencies of IFN-γ-producing CD4+ and CD8 + T cells correlating with the liver injury [61]. The genetic background further modulated these immune responses, potentially explaining how type 1 and type 2 AIH could represent the same disease despite their differing serological profiles in Italian patients with AIH [62]. Thus, the pathogenic mechanisms underlying these clinical differences involved distinct immune responses and genetic profiles that directly influenced transcriptomic signatures. The observed immune dysregulation and cytotoxic T cell signatures in our transcriptomic data may represent a molecular landscape across AIH subtypes and populations.

Moreover, overlap syndromes and atypical presentations increased the complexity of patients with AIH. Autoimmune sclerosing cholangitis (ASC) shared identical serological profiles with AIH-1 (96% ANA/SMA-positive) but exhibited atypical pANCA in 74% of cases [61]. However, the clinical presentation of concurrent autoimmune thyroiditis in AIH patients was often subtle, frequently without overt thyroid dysfunction. Most patients remained euthyroid despite having autoimmune thyroiditis. Studies demonstrated that among pediatric AIH patients with thyroiditis. Studies demonstrated that among pediatric AIH patients with thyroiditis, 60% maintained normal thyroid function, 30% developed subclinical hypothyroidism, and only 10% presented with hyperthyroidism [63]. The spectrum of associated autoimmune conditions extended beyond thyroid involvement to affect multiple organ systems, including primary sclerosing cholangitis, inflammatory bowel disease (ulcerative colitis and Crohn’s disease), rheumatoid arthritis, systemic lupus erythematosus, and celiac disease [64]. This polyautoimmunity resulted from shared immune pathways, genetic predisposition, and environmental triggers that promoted systemic autoimmune susceptibility. The predominant activation of CD8 + T cell, NK cell expansion, and inflammatory cytokine networks we observed may have represented shared immunological pathways underlying multiple autoimmune conditions.

The shared immunological mechanisms resulted in specific and severe clinical manifestations in AIH through key immune pathways. The activation CD8 + T and NK cells in our study may have directly contributed to hepatocellular damage and liver fibrosis through multiple cytotoxic mechanisms. It was shown that CD8 + T cells mediated hepatocyte destruction via programmed cell death pathways, including apoptosis, pyroptosis, and regulated necrosis. Depletion of CD8 + T cells significantly alleviated hepatic inflammation and prolonged survival in the AIH model [65]. This cytotoxic activity was further amplified by the interferon-driven upregulation of MHC class I molecules on hepatocytes, making them more susceptible to CD8 + T cell-mediated killing [66]. CD8 + T cell-mediated emperipolesis directly induced hepatocyte apoptosis in AIH patients, demonstrating their potent cytotoxic capabilities that correlated with histologic severity of liver injury and inflammation [67]. However, the progression from inflammation to fibrosis involved a complex interplay between immune cell dysfunction and profibrotic cellular activation. CXCL10 promoted liver fibrosis by preventing NK cell-mediated inactivation of hepatic stellate cells. In the absence of effective NK cell regulation, activated hepatic stellate cells transform into myofibroblasts, producing excessive collagen and other matrix proteins that led to progressive fibrosis [68]. Based on these mechanistic insights and findings of clinical heterogeneity, personalized treatment strategies should have been implemented to target both the AIH pathology and the concurrent autoimmune manifestations. For patients with highly activated interferon signaling pathways, consideration of targeted interferon antagonists such as anifrolumab should have been prioritized, supported by its demonstrated efficacy in systemic lupus erythematosus and the shared interferon signature between these autoimmune conditions [69]. For partial responders showing impaired CD8 + T cell function and altered Treg/effector T cell ratios, personalized immunosuppressive regimens should have been developed to target specific pathways   [4]. Our findings of immune dysregulation and biomarkers could have enabled prospective risk stratification for early intervention in high-risk patients, potentially allowing anti-fibrotic treatment before irreversible scarring developed. This stratification approach could have been enhanced through NK cell functional assessments and chemokine signaling pathway evaluation [68,70].

This study had several limitations. First, the relatively small sample size limited statistical power, particularly for subgroup analyses of specific clinical phenotypes or concurrent autoimmune conditions, potentially affecting biomarker identification and risk stratification validation. Second, the lack of detailed age distribution data and comprehensive clinical phenotype stratification constrained our ability to perform age-specific analyses and establish phenotype-specific immune signatures. Future studies should involve larger, multicenter cohorts with comprehensive clinical phenotyping to validate these immune dysregulation findings. Finally, while potential treatment biomarkers were identified, the clinical utility of immune cell functional assessments and chemokine signaling pathway evaluations required prospective validation to confirm their predictive value for treatment outcomes and fibrosis progression.

In conclusion, the integrated single-cell and bulk RNA sequencing approach advanced the understanding of AIH pathogenesis. This study identified key immune cell clusters, notably the expansion of CD8 + T cells and NK cells in AIH, along with significant signaling hubs focused on the CCL5-CCR axis. The four immune-related genes and one key transcription factor were screened. These molecular signatures significantly advance our comprehension of AIH immunopathology while simultaneously revealing novel therapeutic targets for precision medicine approaches.

## Supporting information

S1 File Figures. (DOCX)

S1 Table The Bulk DEG from bulk transcriptomic data of GSE159676 and GSE206364. (XLSX)

S2 Table GO enrichment analyses of upregulated genes within the CD8 + T cells, monocyte, and NK cells. (XLSX)

S3 Table GO enrichment analyses of downregulated genes within the CD8 + T cells, monocyte, and NK cells. (XLSX)

S4 Table The ligand-receptor pair pathway in immune cell clusters. (XLSX)
