# Supplementary material for: Decoding the distinct immune landscape and possible regulatory mechanisms of autoimmune hepatitis through integrated single-cell and bulk RNA sequencing
Source: PLoS One. 2025 Dec 4;20(12):e0335605. doi: 10.1371/journal.pone.0335605 (PMC12677505; doi:10.1371/journal.pone.0335605)
Supplement: S1 File — (DOCX) [file pone.0335605.s001.docx]

**Decoding the distinct immune landscape and possible regulatory mechanisms of autoimmune hepatitis through integrated single-cell and bulk RNA sequencing**


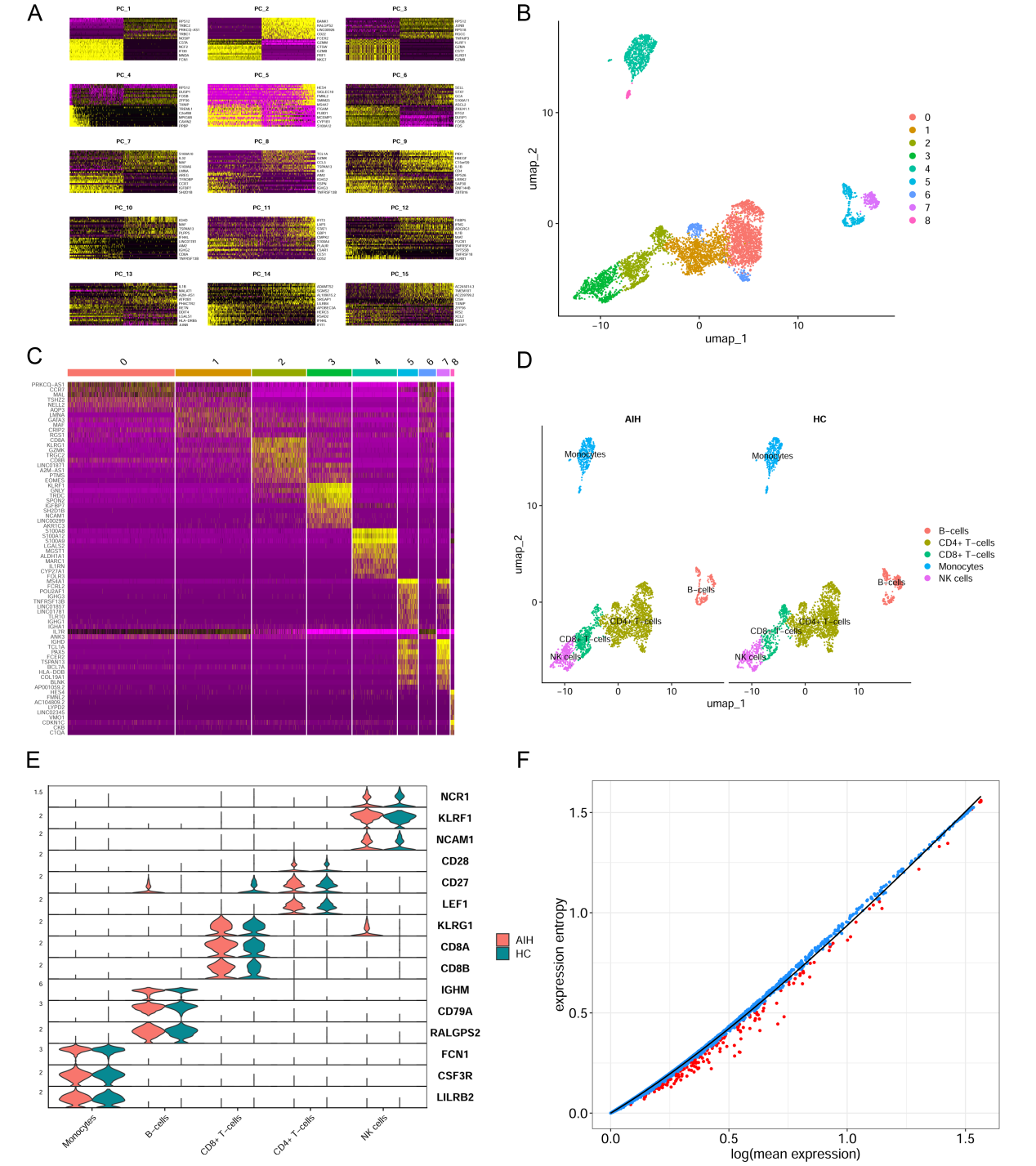


**Figure S1 scRNA analysis in PBMC of autoimmune hepatitis.** (A) Heatmap displaying the top 10 DEGs in each PCA component. (B) UMAP plot showing the identification of nine distinct immune cell clusters. (C) Heatmap of the top 10 DEGs in each immune cell cluster. The top10 DEGs are highlighted in yellow. (D) UMAP projection comparing immune cell cell types between AIH group and HC group. (E) Stacked violin plot depicting the expression of representative marker genes for distinct immune cell types in AIH and HC groups. (F) S-E plot of highly informative genes.


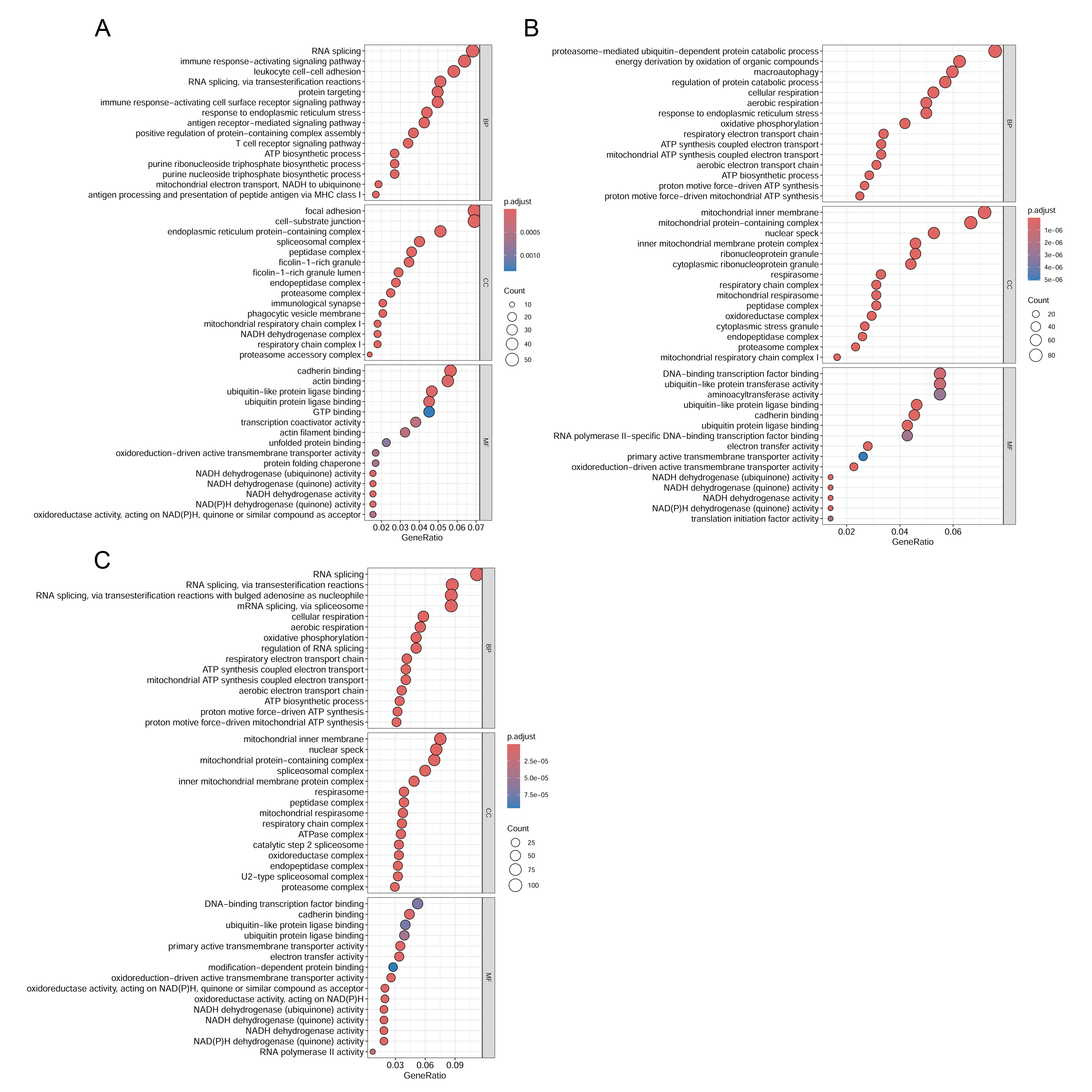


**Figure S2 GO enrichment analysis of downregulated DEGs in the CD8+T cells, monocytes and NK cells from PBMC of autoimmune hepatitis.** (A-C) GO enrichment analysis of downreulated DEGs in CD8+T cells (A), monocytes (B) and NK cells (C).


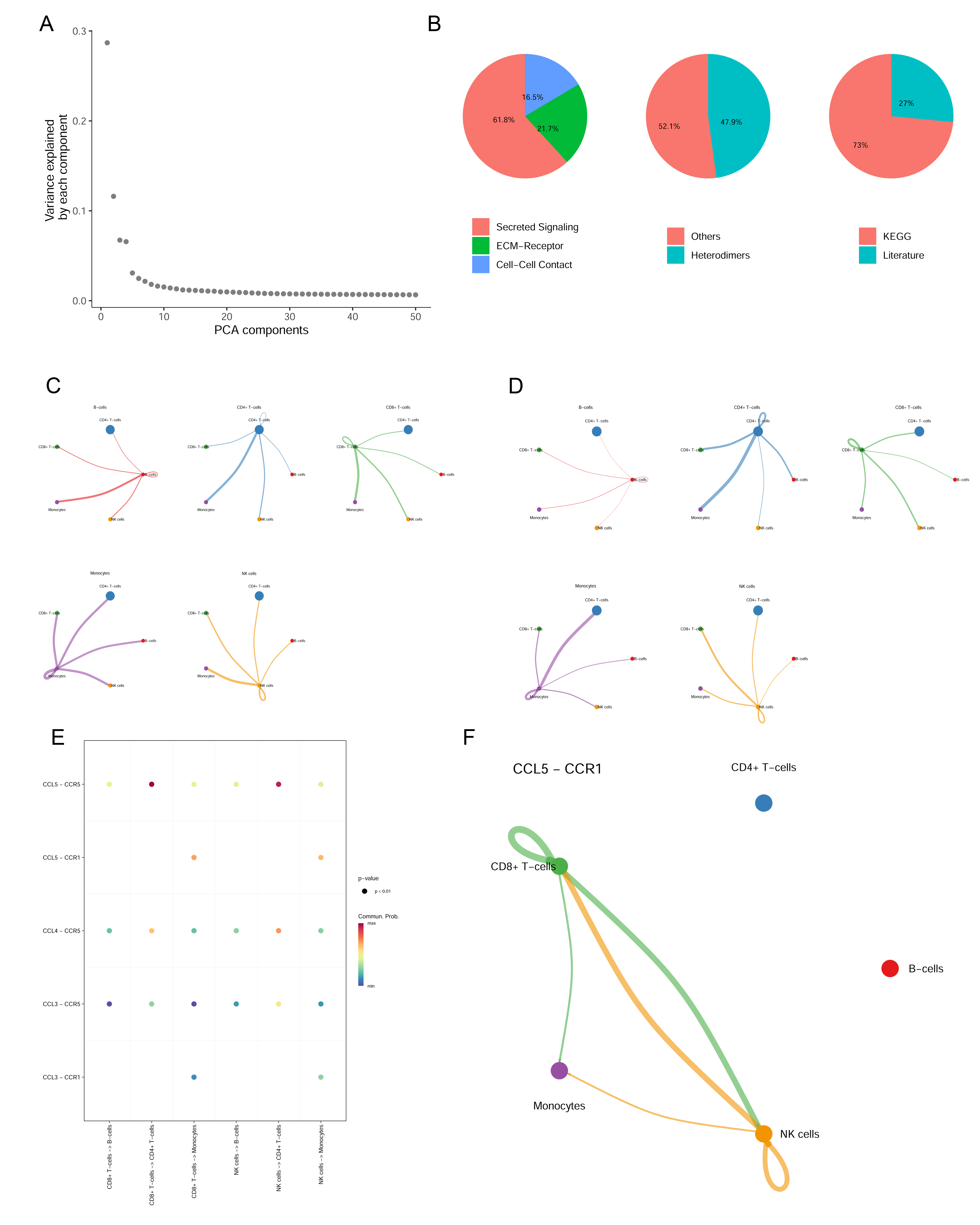


**Figure S3 Cell–cell communication networks analysis in AIH.** (A) Variance explained by each PCA component. (B) Categories in the CellChat database. (C) Number of interactions in each immune cell type. (D) Interaction weights/strength in each immune cell type. (E) Regulatory patterns of the relative communication of chemokine ligand-receptor pairs in different immune cell clusters. (F) Relative strength of CCL5-CCR1 ligand-receptor pair network in immune cell clusters.

**
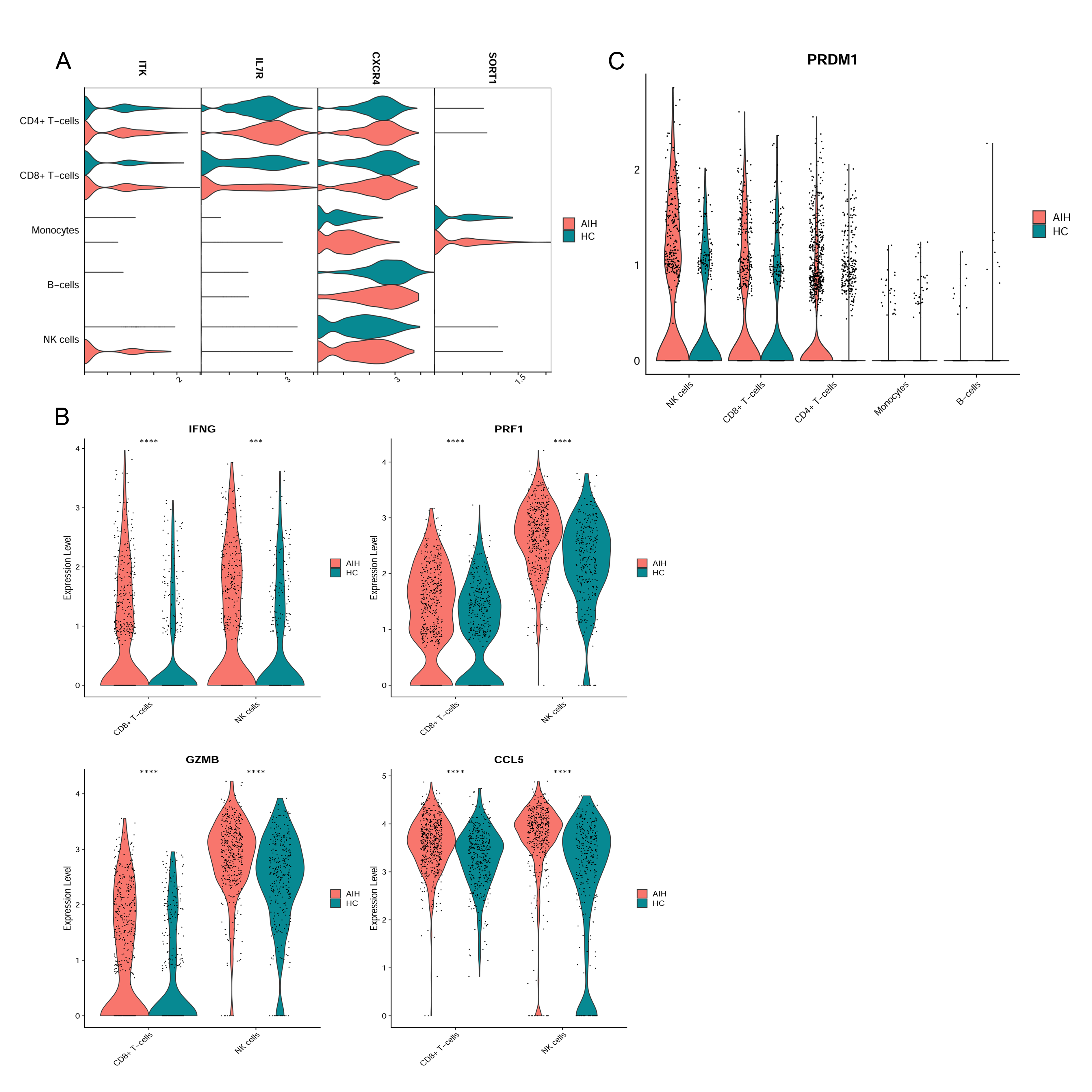
**

**Figure S4 The expression of common DEIRGs and common DETFs from PBMC of autoimmune hepatitis in AIH and HC groups.** (A) Common DEIRGs. (B) The expression of IFN-γ, PRF1, GZMB and CCL5. (C) Common DETFs.
